# Genipin-Enhanced Fibrin Hydrogel and Novel Silk for Intervertebral Disc Repair in a Loaded Bovine Organ Culture Model

**DOI:** 10.3390/jfb9030040

**Published:** 2018-06-24

**Authors:** Daniela A. Frauchiger, Rahel D. May, Ezgi Bakirci, Adel Tekari, Samantha C. W. Chan, Michael Wöltje, Lorin M. Benneker, Benjamin Gantenbein

**Affiliations:** 1 Tissue & Organ Mechano Biology, Institute for Surgical Technology and Biomechanics, University of Bern, 3012 Bern, Switzerland; daniela.frauchiger@istb.unibe.ch (D.A.F.); Rahel.May@istb.unibe.ch (R.D.M.); ezgibakirci@gmail.com (E.B.); adel.tekari@istb.unibe.ch (A.T.); samanthacn@gmail.com (S.C.W.C.); 2 Laboratory of Molecular and Cellular Screening Processes, Centre of Biotechnology of Sfax, University of Sfax, Sfax 3018, Tunisia; 3 Institute of Textile Machinery and High Performance Material Technology, TU Dresden, 01062 Dresden, Germany; michael.woeltje@tu-dresden.de; 4 Department of Orthopaedic Surgery and Traumatology, Spine Unit, Insel Hospital, Bern University Hospital, Bern 3010, Switzerland; lorin.benneker@insel.ch

**Keywords:** organ culture, bioreactor, intervertebral disc, mechanical loading, genipin, fibrin, silk, repair, fibre-reinforced hydrogel, qPCR, cell activity, histology

## Abstract

(1) Background: Intervertebral disc (IVD) repair represents a major challenge. Using functionalised biomaterials such as silk combined with enforced hydrogels might be a promising approach for disc repair. We aimed to test an IVD repair approach by combining a genipin-enhanced fibrin hydrogel with an engineered silk scaffold under complex load, after inducing an injury in a bovine whole organ IVD culture; (2) Methods: Bovine coccygeal IVDs were isolated from ~1-year-old animals within four hours post-mortem. Then, an injury in the annulus fibrosus was induced by a 2 mm biopsy punch. The repair approach consisted of genipin-enhanced fibrin hydrogel that was used to fill up the cavity. To seal the injury, a Good Manufacturing Practise (GMP)-compliant engineered silk fleece-membrane composite was applied and secured by the cross-linked hydrogel. Then, IVDs were exposed to one of three loading conditions: no load, static load and complex load in a two-degree-of-freedom bioreactor for 14 days. Followed by assessing DNA and matrix content, qPCR and histology, the injured discs were compared to an uninjured control IVD that underwent the same loading profiles. In addition, the genipin-enhanced fibrin hydrogel was further investigated with respect to cytotoxicity on human stem cells, annulus fibrosus, and nucleus pulposus cells; (3) Results: The repair was successful as no herniation could be detected for any of the three loading conditions. Disc height was not recovered by the repair DNA and matrix contents were comparable to a healthy, untreated control disc. Genipin resulted being cytotoxic in the in vitro test but did not show adverse effects when used for the organ culture model; (4) Conclusions: The current study indicated that the combination of the two biomaterials, i.e., genipin-enhanced fibrin hydrogel and an engineered silk scaffold, was a promising approach for IVD repair. Furthermore, genipin-enhanced fibrin hydrogel was not suitable for cell cultures; however, it was highly applicable as a filler material.

## 1. Introduction

Currently, the elderly population is increasingly affected by lower back pain. This condition causes a tremendous burden on the economy, society, and the individuals who suffer from it [1]; hence, better treatment options are in high demand. Often, the pain is caused by disc herniation, where the protruding gelatinous core of the intervertebral disc (IVD), the nucleus pulposus (NP), might cause inflammation or pressure on a nerve, which causes pain. Another scenario is disc degeneration, where, over time, the NP becomes less hydrated, and the encompassing lamellar structure, the annulus fibrosus (AF), shows fissure formation. This may lead to disc height loss, which can cause pressure on the nerves or scuffing of the facet joints; both of which may lead to paralysis or pain. Nevertheless, today’s gold standard treatment options, including medication and surgical interventions, in most cases do not target the source of the pain. Actually, these options only focus on alleviating the symptoms and temporarily relieving the pain.

In this manuscript, we focus on repairing the AF after disc herniation with the combination of specifically adjusted biomaterials for IVD repair. This form of treatment is gaining in importance and is increasingly investigated [2]. Recently, AF injury was induced in vivo in a sheep model by injection of riboflavin cross-linked high-density collagen gel ability to mitigate IVD degeneration after induced AF injury [3].

One of the materials particularly suggested for AF repair is a genipin-enhanced fibrin hydrogel, which has become promising not only for IVD but also for cartilage repair [4] as it can fix an AF injury such that it can withstand applied compressional and torsional loads [2,5]. Fibrin hydrogels have already been widely used for years as a sealant for the AF, the outer tissue within the IVD, during surgeries but are too fragile in this form to withstand physiological loading in the spine. However, the stiffness can be increased by the addition of genipin to mimic the mechanical properties of the AF [6,7].

Silk is an uttermost exciting biomaterial and has been proposed in orthopaedics for many applications including bone-, anterior cruciate ligament-, tendon-, meniscus-, and IVD repair [8,9,10,11]. Silk keeps in the research focus since it is resorbable, fine-tuneable for slow release of incorporated growth factors [12] and can be liquefied or applied in different knitted forms or also in modern concepts of [8,13,14,15]. Silk has outstanding tensile strengths and low inflammatory response characteristics if applied degummed from sericin [16,17,18].

In order to tackle the many requirements of IVD repair, i.e., six degrees of freedom loading, avascularity, and low cell density, a single biomaterial might not be sufficient. Hence, a genipin-enhanced fibrin hydrogel was combined with different materials and substances to achieve optimal functionality [2,5,19]. Another problem faced in IVD repair is the evaluation of such a therapy in vitro and ex vivo. Here, the use of bioreactors has moved into the spotlight over the last decade [20]. They allow for the investigation of preliminary ideas in not only a less complex manner but also in a more affordable way than in vivo experiments. Furthermore, bioreactor experiments do often not require an ethical permit, as the IVDs of the animals originate from the food chain. Moreover, by using animal models with bioreactors, it is possible to assess the baseline, i.e., healthy properties of the IVD and to compare it under comparable conditions to the other experimental groups. Today’s bioreactors are capable of applying compression (static or dynamic) on animal IVDs, e.g., bovine and ovine [20,21,22,23]. For human samples, two bioreactors for compressional loading of lumbar IVDs were designed lately [22,24]. Nevertheless, only one can apply torsion in addition to compression to mimic physiological loading over a prolonged time on living IVDs [25].

In this study, we aimed to shed light on an inside-out repair approach combining two biomaterials in a clinically relevant setting by using a physiological bovine organ culture model. We hypothesized that the combination of a custom-engineered *Bombyx mori* silk membrane-fleece and a genipin-enhanced fibrin hydrogel could repair a large AF injury, comparable to disc herniation, in terms of restoring disc height and biological function. After inducing a 2 mm circular injury with a biopsy punch, a genipin-enhanced human fibrin hydrogel was used as a filling agent and the silk membrane-fleece as a sealant to repair the injury. The repaired IVDs were then compared to the injury only group and normalized to healthy control IVDs. The three experimental groups were subjected to either no loading, static loading or complex loading conditions [12]. To mimic physiological loads, complex loading was performed in a two-degree-of-freedom bioreactor that allows for compression and torsion [25].

## 2. Results

### 2.1. Stress-Strain Measurement

**Figure 1:**
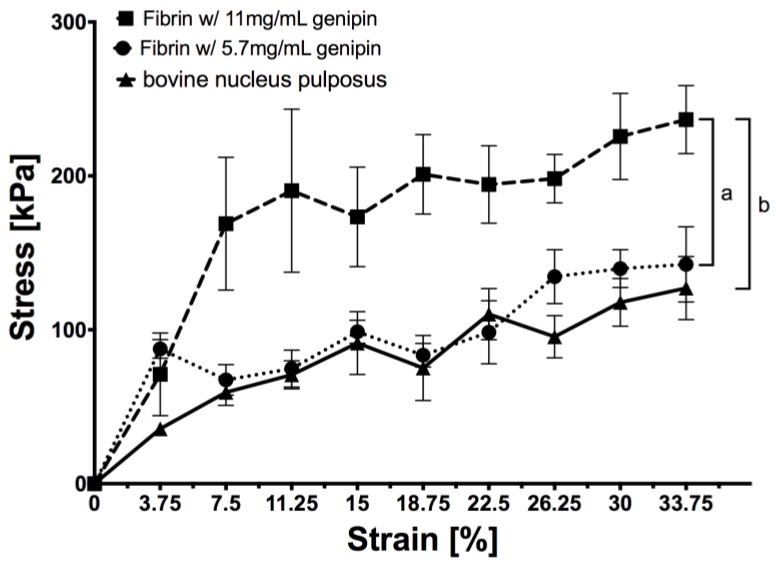
Stress-Strain curves for two genipin-enhanced fibrin hydrogels (5.7 and 11 mg/mL fibrin) and bovine nucleus pulposus tissue. Nominal compressive stress was calculated from force measurements of 6 mm wide and 4 mm high circular discs under unconfined conditions in pre-warmed PBS, N = 5, mean ± SEM, two-way ANOVA: *p*-value *a* < 0.05, *b* < 0.001.

Both genipin-enhanced fibrin hydrogels and the bovine NP (bNP) tissue could be tested up to 37.5% of strain (Figure 1). When comparing them, no statistical difference was found for bNP and 5.7 mg/mL genipin (two-way ANOVA *p* > 0.1) whereas 11 mg/mL genipin resulted in a significantly stiffer hydrogel compared to bNP (*p* < 0.01) and 5.7 mg/mL genipin (*p* < 0.05).

### 2.2. Cytotoxicity of Genipin

**Figure 2:**
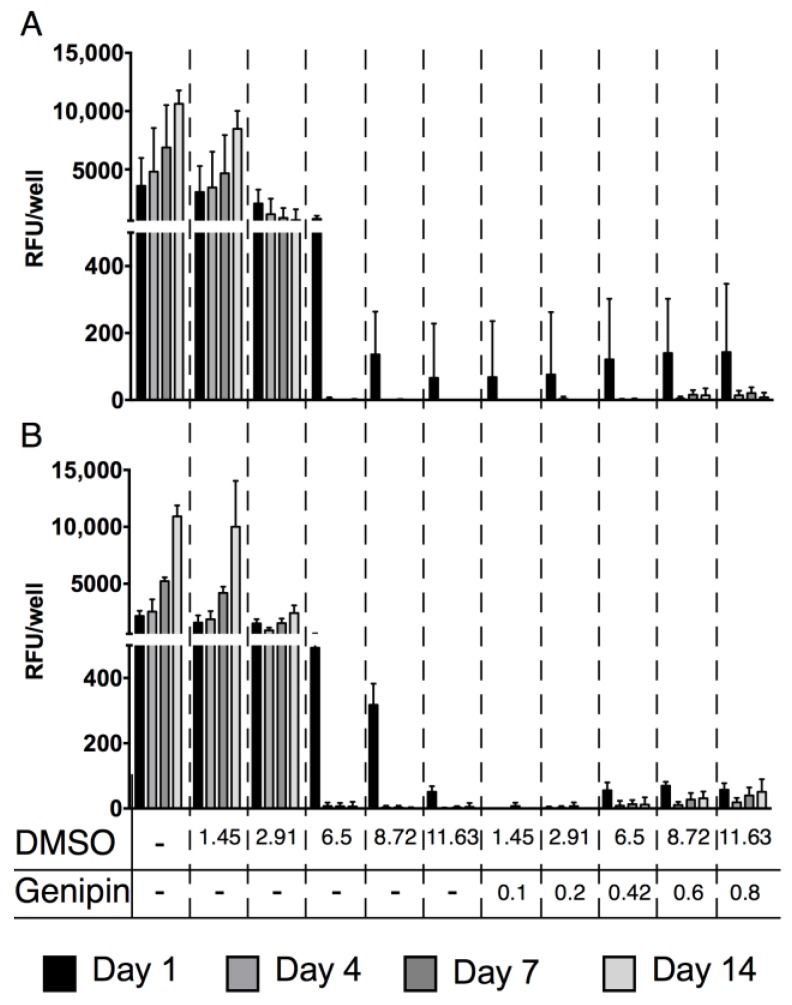
Mitochondrial activity of genipin cytotoxicity assay. (**A**) human mesenchymal stem cells and (**B**) human annulus fibrosus and nucleus pulposus cells were mixed with increasing dimethyl sulfoxide (DMSO) and genipin concentration and the mitochondrial activity was determined after 1, 4, 7 and 14 days of culture.

For all human cell types tested (human AF cells [hAFC], human NP cells [hNPC] and human mesenchymal stem cells [hMSC]), increasing dimethyl sulfoxide (DMSO) concentrations impaired the cells’ mitochondrial activity. These cells could only cope with the lowest DMSO concentrations (mean ± SEM, hMSC: control day one 3589 ± 976 vs. day 14 10,633 ± 572, 1.45% DMSO day one 3052 ± 916 vs. day 14 8481 ± 770, 2.91% DMSO day one 2069 ± 491 vs. day 14 655 ± 413; hAFC and hNPC: control day one: 2154 ± 188 vs. day 14 10,919 ± 431 RFU, 1.45% DMSO day one 1581 ± 256 vs. day 14 10,008 ± 1649 RFU, 2.91% DMSO day one 1500 ± 152 vs. day 14 2423 ± 274 RFU). For higher DMSO concentrations (6.5% and higher), no mitochondrial activity was detected. Furthermore, when combined with genipin, even at the lowest DMSO/genipin concentrations, no mitochondrial activity could be measured (hMSC: 0.1% genipin day one 68 ± 68 vs. day 14 0 ± 0, hAFC and hNPC: 0.1% DMSO day one 0 ± 0 vs. day 14 0 ± 0 RFU) (Figure 2).

### 2.3. Organ Culture

**Figure 3:**
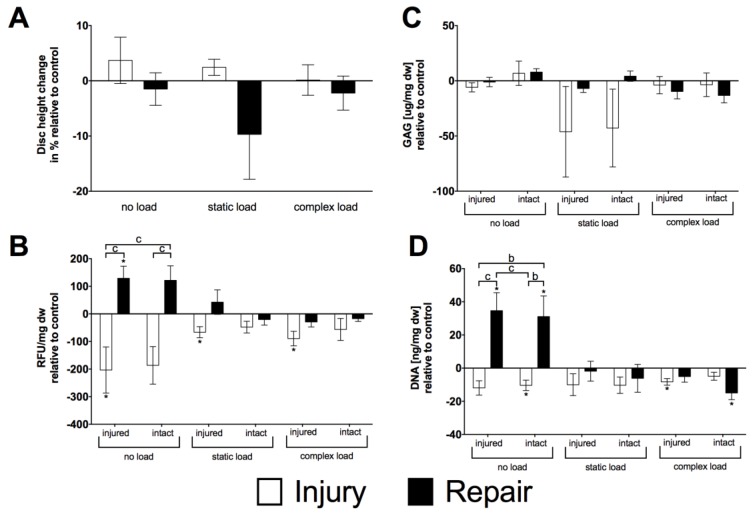
(**A**) Disc height; (**B**) mitochondrial activity; (**C**) GAG content normalized to dry weight relative to control discs; (**D**) DNA content normalized to dry weight and relative to control disc. Mean ± SEM, N = 5, *t*-test: *p*-value * < 0.05; two-way ANOVA: *p*-value *b* < 0.01, *c* < 0.001.

After two weeks of organ culture, the initial disc height was compared to the final disc height relative to respective control IVDs. There was no significant main effect for treatment, i.e., injury and repair (two-way ANOVA *p* > 0.05), neither for the main effect of the loading condition (two-way ANOVA *p* > 0.1) (Figure 3A). Despite not seeing differences regarding disc height, mitochondrial activity differed significantly (two-way ANOVA *p* <0.001) between injury and repair model for both locations on the disc, injured (injury: −203.44 ± 83.64 RFU/mg dw vs. repair: 128.99 ± 44.02 RFU/mg dw) and intact (injury: −186.58 ± 68.08 RFU/mg dw vs. repair: 121.48 ± 52.66 RFU/mg dw) in the case of no load. Except the samples taken next to the injury (injured) for the injury samples (static load: −66.42 ± 20.10 RFU/mg dw, complex load: −89.58 ± 26.27 RFU/mg dw; *p* < 0.05), static and complex loaded samples were similar for both the repair and injury model, being in the range of the healthy control disc (Figure 3B). This observation was in accordance with the measured DNA contents. Also, here, the repaired discs outperformed the injury disc in the no load organ culture (two-way ANOVA *p* < 0.001; injury model: intact: −10.39 ± 3.14 ng/mg dw, injured: −11.93 ± 4.31 ng/mg dw, repair model: intact: 31.09 ± 12.44 ng/mg dw, injured: 34.63 ± 10.84 ng/mg dw) (Figure 3D). Nevertheless, this same behaviour could not be observed for matrix production. Here, glycosaminoglycans (GAG) content remained unchanged in the range of the control discs; i.e., for no load injury model: intact 6.78 ± 11.02 µg/mg dw, injured: −5.93 ± 4.14 µg/mg dw, repair model: intact: 7.88 ± 3.05 µg/mg dw, injured: −1.12 ± 4.25 µg/mg dw, static load injury model: intact: −42.79 ± 35.22 µg/mg dw, injured: −46.17 ± 41.06 µg/mg dw, repair model: intact: 4.18 ± 4.65 µg/mg dw, injured: −6.97 ± 3.61 µg/mg dw, injured −3.95 ± 7.81 µg/mg dw, repair model: intact: −13.26 ± 6.72 µg/mg dw, injured: −9.66 ± 6.78 µg/mg dw (two-way ANOVA *p* > 0.05) (Figure 3C).

### 2.4. Gene Expression

**Figure 4:**
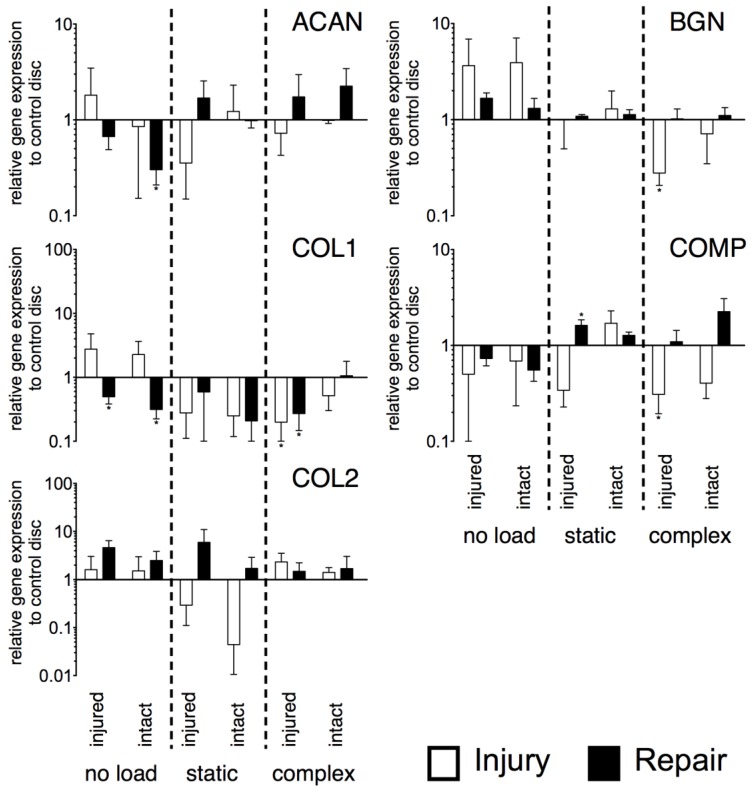
Relative expression of major anabolic genes. N = 6, mean ± SEM, *p*-value * < 0.05.

Gene expression of anabolic genes (*aggrecan* [*ACAN*], *collagen type I* [*COL1*] and *type II* [*COL2*], *biglycan* [*BGN*] and *cartilage oligomeric matrix protein* [*COMP*]) did not show significant differences between the injured and repaired IVDs under the three loading conditions (Figure 4). Furthermore, no difference from the control discs was determined for *COL2*. For *ACAN*, significant down-regulation was found for the repaired disc on the intact side under no load (*p* < 0.05, ~3-fold). The expression of *COL1* was down-regulated for both sides of the repaired discs under no load (injured: *p* < 0.05, ~2-fold; intact: *p* < 0.05, ~3-fold). Furthermore, both IVDs, i.e., injured and repaired, were down-regulated under complex load on their injured side (injury: *p* < 0.05, ~5-fold, repair: *p* < 0.05, ~4-fold). Also, *BGN* showed down-regulation for the injured disc under complex load on the injured side (*p* < 0.05, ~5-fold). Finally, *COMP* was also down-regulated for the injured side of the injured disc under complex load (*p* < 0.05, ~5-fold) whereas under static load, an up-regulation of *COMP* was observed for the repaired disc on the site of repair (*p* < 0.05, ~2-fold).

**Figure 5:**
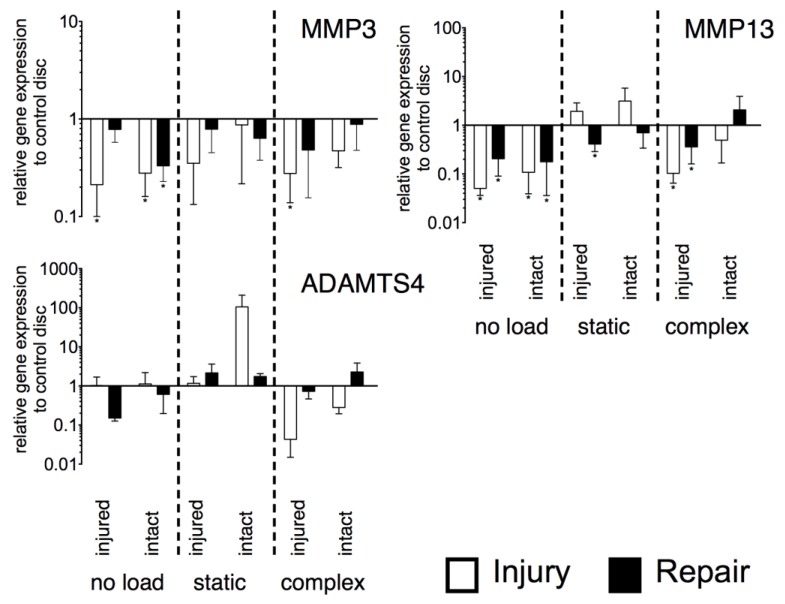
Relative expression of major catabolic genes. N = 6, mean ± SEM, *p*-value * < 0.05.

As observed for the anabolic genes, also the catabolic genes (*matrix metallopeptidase 3* [*MMP3*] and *13* [*MMP13*], *ADAM metallopeptidase with thrombospondin type 1 motif 4* [*ADAMTS*4]) did not show significant differences between the repaired and injured IVDs (Figure 5). For *MMP3* and *MMP13*, significant down-regulation was observed mainly for the unloaded samples whereas *ADAMTS4* did not show significant changes from the control discs. Expression of *MMP3* was down-regulated on both sides of the injured disc under no load (injured: *p* < 0.05, ~5-fold, intact: *p* < 0.05, ~4-fold) and on the injured side under complex load (*p* < 0.05, ~4-fold). For the repaired IVDs, down-regulation was only observed on the intact side of unloaded samples (*p* < 0.05, ~3-fold). Under no load, *MMP13* was down-regulated for both sides of the injured (injured: *p* < 0.05, ~20-fold; intact: *p* < 0.05, ~9-fold) and repaired IVDs (injured: *p* < 0.05, ~5-fold; intact: *p* < 0.05, ~6-fold). Also, under complex load, the injured side of the injured (*p* < 0.05, ~10-fold) and repaired IVDs (*p* < 0.05, ~3-fold), *MMP13* was down-regulated, whereas under static load down-regulation was only observed for the injured side of the repaired IVDs (*p* < 0.05, ~2-fold).

**Figure 6:**
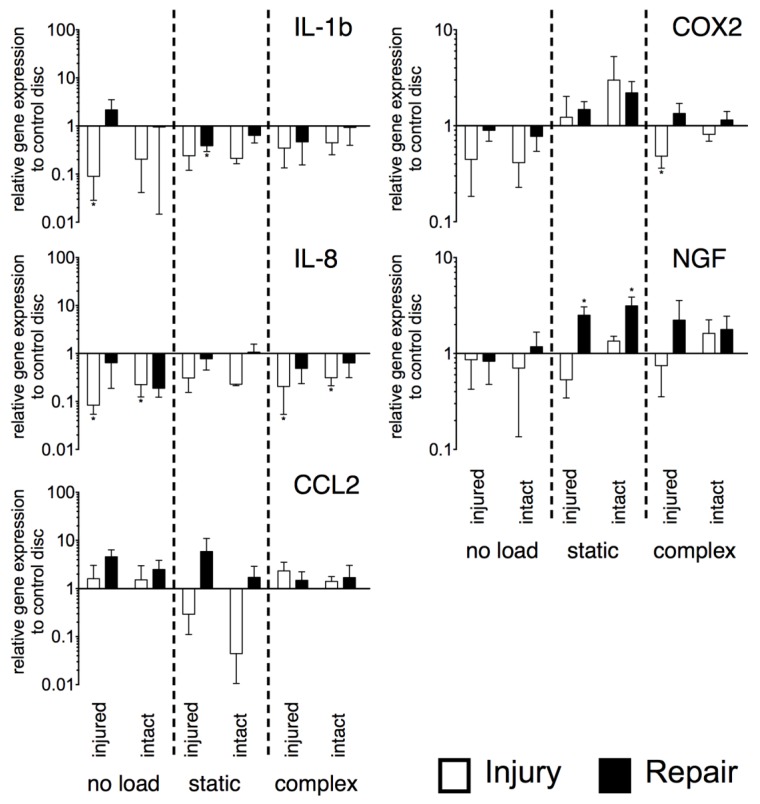
Relative expression of major inflammatory genes. N = 6, mean ± SEM, *p*-value * < 0.05.

Also, the inflammatory genes tested (*interleukin 1 beta* [*IL-1b*] and *8* [*IL-8*], *chemokine (C-C motif) ligand 2* [*CCL2*], *cyclooxygenase-2* [*COX*2], and *nerve growth factor* [*NGF*]) showed no significant differences between injured and repaired IVDs (Figure 6). However, significantly different gene expression from control IVDs were observed. *IL-1β* was down-regulated under no load for the injured side of the injured IVDs (*p* < 0.05, ~11-fold). Furthermore, under static load, down-regulation was found for the repaired disc on the injured side (*p* < 0.05, ~3-fold). For *IL-8*, both sides of the injured IVDs showed down-regulation under no load (injured: *p* < 0.05, ~12-fold; intact: *p* < 0.05, ~4-fold) and under complex load (injured: *p* < 0.05, ~5-fold; intact: *p* < 0.05, ~3-fold). Expression of *COX2* was down-regulated for the injured side of the injured IVDs under complex load (*p* < 0.05, ~2-fold). *NGF* was up-regulated under static load for both sides of the repaired IVDs (injured: *p* < 0.05, ~3-fold, intact: *p* < 0.05, ~3-fold). For *CCL2*, gene expression did not differ from healthy control IVDs.

### 2.5. Histology

**Figure 7:**
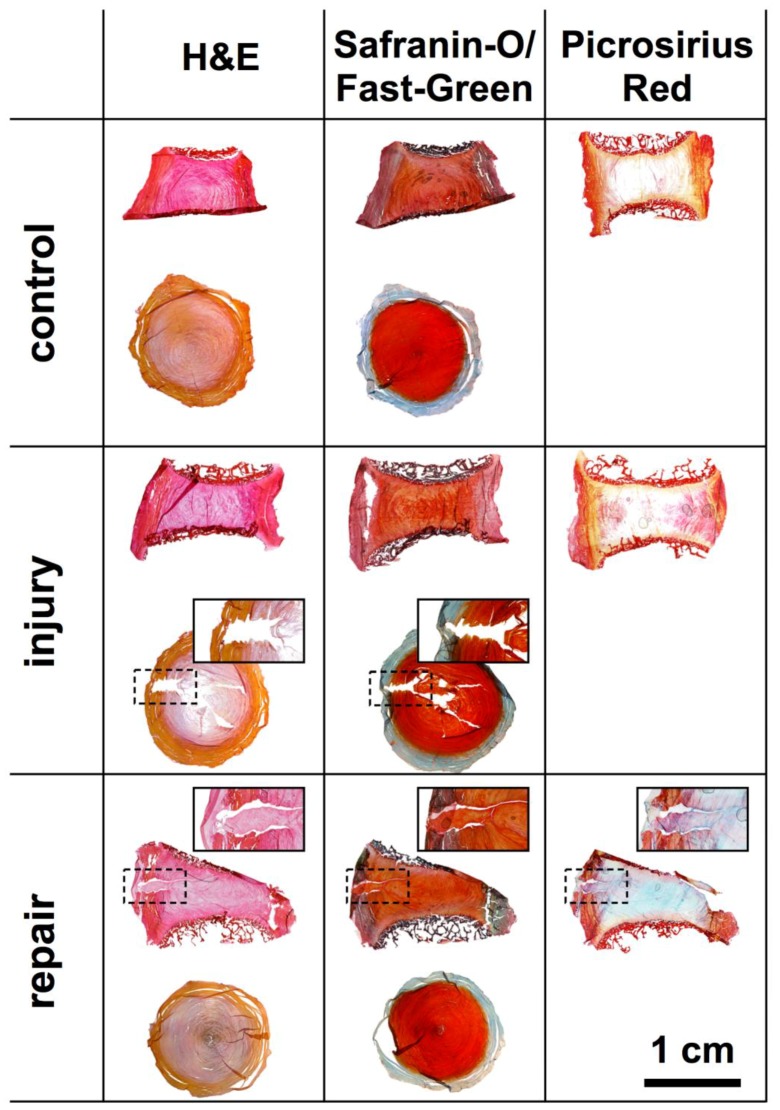
Histology of sagittal PMMA and transversal cryosections of IVDs after 14 days of culture under free-swelling conditions. (**Left**–**Right**) Hematoxylin & Eosin (H & E), Safranin-O/Fast Green and Picrosirius Red. (**Top**–**Down**) Healthy control, injury and repair discs.

Histological sections showed the sites of injury and repair (Figure 7). The biopsy punch injury created a trajectory that was easily detectable in the transversal cryosections. However, in the sagittal PMMA sections, the injury was not visible. No difference that would suggest the loss of GAGs could be seen after 14 days of culture in histological sections. The repair was only visible in the sagittal sections, where the open space created by the injury is filled up by the genipin-enhanced fibrin hydrogel. Nevertheless, a small gap, separating the hydrogel and native IVD tissue was visible in the sections. Furthermore, as an artefact of the preparation the silk did not remain on the disc throughout sample preparation; however, the hydrogel applied as a filler and glue for the silk, remained intact and formed a tight seal (Figure 7).

## 3. Discussion

### 3.1. IVD Injury versus Repair

The IVD repair approach presented here combined a genipin-enhanced fibrin hydrogel with a novel engineered silk fleece-membrane composite. The repair was successful over two weeks under all loading conditions tested as the hydrogel was capable of withstanding the forces applied and was able to hold the silk fleece-membrane composite in place. Furthermore, no herniation was observed with none of the loading regimes. The disc height increase observed in the injury group possibly was caused by the better accessibility of medium to the NP and hence an increased swelling. Whereas, in the repaired IVD medium diffusion was only possible physiologically into the disc and hence uncontrolled swelling of the tissue was prevented. Hence, disc height was not restored in repaired IVDs; however, this did not negatively affect DNA and GAG contents (Figure 3). It seemed that preventing uncontrolled swelling of the IVD acted beneficial onto cell survival (mitochondrial activity) and DNA content also GAG content did not differ significantly from control. For the injured discs under static loading, a trend towards a decrease in GAG content was detected, which might indicate an irreversible recovery of the disc due to the invasive induced injury. In this case, despite the better match between 5.7 mg/mL genipin-enhanced fibrin hydrogel and bNP tissue, the higher genipin concentration might have been better suited to prevent GAG loss. Nevertheless, introducing a too-stiff material into the NP might probably result in other negative effects on cell survival in the IVD.

Relative gene expression of both sides (injured vs. intact) of the IVD showed in most cases a comparable level among all the genes monitored. We attributed this effect to the particular static culture condition used as the IVDs released cytokines due to the injury diffused into the medium and also affected the contralateral side of the IVD. Gene expression did not reveal significant differences between the injured and the repaired IVD, and was predominantly in the range of the healthy control discs. This was in accordance with GAG contents that were also not significantly different from the control IVDs. Furthermore, inflammatory marker genes mainly did not differ significantly from healthy control discs. Only a significant increase in NGF for statically loaded repaired discs was found, which might be responsible for innervation and was often associated with painful discs [26]. Other approaches of IVD damage involving AF damage and disc repair models were lately tested in vivo ovine using defined drill bit injuries induced to the AF [27]. It would be interesting to see how a composite approach using the silk-membrane fleece and fibrin hydrogel for such a model would perform [3]. Very important are, of course, the duration of studies and follow-up. In vivo ovine models being inferior in terms of presence of immune cells and more human-like conditions for IVD repair.

### 3.2. Toxicity of Genipin

The promising mechanical properties of adding genipin, which increased the stiffness of the hydrogel were overshadowed by the strong cytotoxic effects after only 24 h of exposure for all cell types tested (Figure 3). This difference from the 3D culture might arise from the simplified 2D approach that was chosen after encountering difficulties in the evaluation of live/dead stain due to autofluorescence in 3D scaffolds. Nevertheless, the investigation of mitochondrial activity from organ culture experiments revealed no cytotoxic effect of genipin-enhanced fibrin hydrogel to the surrounding tissue. This behaviour was also observed by Guterl et al. (2014) [28] when cells were seeded onto cross-linked genipin-enhanced fibrin hydrogel carriers. Furthermore, a degradation study of subcutaneously injected genipin-enhanced fibrin hydrogel showed cell infiltration and degradation over 16 weeks [6]. Therefore, the superior mechanical properties make genipin-enhanced fibrin hydrogel an ideal candidate for an IVD filler material to re-establish the IVDs’ mechanical properties combined with the engineered silk fleece-membrane as tested in this study. However, the establishment of reliable 3D cell viability methods to judge the cytotoxicity in 3D genipin-hydrogels using live/dead stain was not possible due to the strong auto-fluorescence behaviour of the genipin during laser microscopy [29].

If looking at histology, no integration of the hydrogel with the native tissue occurred (Figure 7). Hence, no cell migration into the hydrogel could be observed. Nevertheless, the injury was tightly closed by the hydrogel and silk fleece-membrane. Moreover, the hydrogel in the wound trajectory prevented the NP from protruding completely into the AF region. Furthermore, also here, no adverse effects due to the genipin were observed. Additionally, the silk composite might be used to culture or condition cells prior AF repair, when engineered to contain growth factors such as GDF-6 [12].

### 3.3. Limitations and Strengths

Strengths

- The presented IVD damage and repair model allowed for a highly controlled culture environment and using well-controlled diurnal dynamic loading parameter conditions [21,30].
- A constructed GMP-compliant silk-fleece material was able to seal the outer AF and stayed in place over 14 days of repetitive mechanical loading using a fibrin hydrogel as a filler material.
- The addition of the natural cross-linker genipin resulted in a significantly stiffer hydrogel coming close to the native bovine NP tissue [6,31].

Limitations

- Cytotoxic assays of genipin in monolayer assays demonstrated a high cytotoxicity, representing hurdles for the American Food and Drug Administration (FDA) or the Conformité Européene (CE) approval for future research towards clinical application.
- The ex vivo organ culture model to test the effect of biomaterials onto the cell viability/cell activity was limited to 14 days due to the chosen bovine model with endplates attached, which limited the diffusion of glucose or other unknown factors over longer culture periods. Here, application of improved preparation techniques and culture media might allow to prolong the culture period and mechanical loading [32].

### 3.4. Future Road Map for Fine-Tuning Hydrogels and/or Silk

The current organ culture study demonstrated the potential of combining biocompatible hydrogels, which are often too soft but with added cross-linkers that can functionalize stiffness to a desired application such as IVD repair. Bovine coccygeal IVDs are currently proposed as an easily accessible source to test spinal repair scenarios [33]. Of course, there are anatomical differences to be considered to the human IVD and the fact that facet joints and muscle connections had to be omitted [24]. The biopsy punch injury model has been proposed now by two recent studies [34,35], which resulted in about 50% penetration depth of the IVD diameter [35]. It is currently unknown how bigger damage models would respond when even >50% or more of the tissue was penetrated and mechanically removed. It has been shown that mechanical overloading will induce stress and increased cell death [36] in the short term (weeks to months). However, it is currently unknown whether AF ruptures [37] would cause an even bigger damage if primed initially and then followed by physiological or hyper-physiological complex loading [38]. Future clinically relevant investigations would also be needed to compare the shifting in fibre structure of a re-enforced hydrogel depending on the number of dynamic loading cycles under compression and torsion.

## 4. Material and Methods

### 4.1. IVD Isolation and Culture

**Figure 8:**
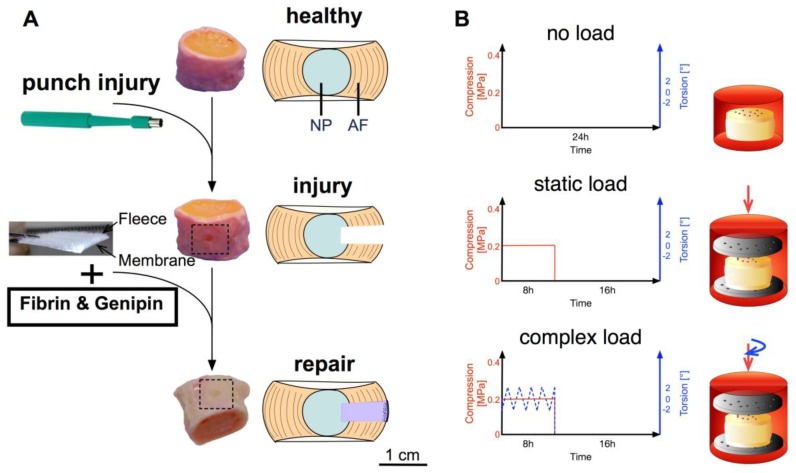
(**A**) Experimental design from healthy to injured to repaired disc. An injury was induced by a 2 mm biopsy punch. The cavity was filled with a genipin-enhanced fibrin hydrogel and closed with an engineered silk-fleece composite with the fleece side facing to the IVD. (**B**) Loading profiles used for 14 days of organ culture either no load, static load (0.2 MPa for 8 h/d) or complex load (0.2 MPa and 0 ± 2° of torsion at 0.2 Hz both for 8 h/d).

Coccygeal bovine IVDs were isolated from 10- to 14-months-old animals that were obtained from a local abattoir a few hours after slaughter as described by Chan et al. (2012) [39]. In short, skinned tails were immersed in a betadine solution for disinfection. Then under aseptic conditions, IVDs were exposed by removing muscle, fat and tendon tissue. Single IVDs were excised by using a custom made cutting tool along the endplate of the IVD. Followed by cleaning the endplate with Ringer solution and a jet lavage system to remove blood clots and ensure passage of nutrients into the IVD through the endplate. After isolation, the IVDs were equilibrated in free-swelling conditions for ~17 h in high-glucose Dulbecco’s modified Eagle’s medium (HG-DMEM) (Gibco, Thermo Fisher Scientific, Zug, Switzerland) supplemented with 5% fetal calf serum (FCS) and 1% penicillin/streptomycin. On the following day, the IVDs were then split into three experimental groups: (i) either left untreated (control); (ii) injured or (iii) an injury followed by the repair. IVDs were then cultured for 14 days under three different loading conditions: (1) free swelling where the discs were kept in HG-DMEM; (2) static load with 0.2 MPa compression for 8 h/day; and (3) complex loading with 0.2 MPa compression superimposed with torsion of 0 ± 2° at a frequency of 0.2 Hz. For the application of the complex load, a custom-designed, two-degree-of-freedom bioreactor was used (Figure 8B) [30].

### 4.2. Injury and Repair Model

IVD injury was induced through the application of a single 2 mm circular biopsy punch, with the centre being removed with a scalpel to create a cavity [34]. The hydrogel was prepared as described by Guterl et al. (2014) [28] and then adjusted to our needs as follows: Human-based fibrinogen (~45 mg/mL, Tisseel, Baxter, Glattpark, Switzerland) was supplemented with FCS and 𝜀-aminocaproic acid [40]. Prior to addition to the fibrinogen, the genipin (5.7 mg/mL fibrinogen, Waco Chemicals GmbH, Neuss, Germany) was dissolved in DMSO and mixed thoroughly with thrombin (10 U/mL, Tisseel, Baxter, Deerfield, IL, USA). The cavity of half of the injured discs was filled with the hydrogel and a silk-scaffold (Spintec Engineering GmbH, Aachen, Germany) that was used to cover the cavity with the fleece side facing the disc (Figure 8A). The silk-scaffolds were produced from *Bombyx mori* larvae that were grown under sterile conditions. Silk fibroin was harvested directly from the silk glands to minimize contamination with sericin, what otherwise might cause allergic reactions. Details of the production of the silk-scaffold used can be found in Frauchiger et al. (2017) [12]. After 15 min of cross-linking, the IVDs were transferred to the FCS-containing medium, and the respective loading profile was started. Data were normalized to untreated healthy control and compared to injury only IVDs, both underwent the same loading profile as the repaired IVDs (each N = 5).

### 4.3. Stress–Strain Measurement

Hydrogel discs were prepared in silicon moulds with diameters of 6 mm, heights of 4 mm, and volumes of 150 µL. Fibrin hydrogel was prepared using a commercial kit by Baxter (Tisseel, Baxter). Fibrinogen was diluted to obtain ~45 mg/mL of protein, and the cross-linking reaction was started by addition of thrombin (10 U/mL) mixed with either 5.7 mg/mL or 11.4 mg/mL genipin. Fibrin hydrogels (N = 5) were tested after twelve hours of incubation at room temperature (RT) in a humid atmosphere to prevent dehydration of hydrogel. Additionally, five samples were prepared from one bovine NP of 6 mm width and ~3–5 mm in height. These samples were used to compare the mechanical properties of the hydrogels to the natural condition of the disc. For measurements, a mini-bioreactor was used as previously described [41], where after initial contact, the strain was increased by 3.75% every two minutes up to 37.5% of strain. The maximum force upon strain increase was used to calculate nominal compressive stress. The experiments were performed under unconfined compression at RT and in pre-warmed PBS (37 °C) to prevent dehydration of hydrogels and to mimic the situation in vivo.

### 4.4. Cytotoxicity of Genipin

Human MSC (hMSC), AF cells (AFC), and NP cells (NPC) were isolated from bone marrow and disc tissue, respectively (Table 1). Procedures are ethically approved by the ethics committee of the canton of Bern and tissues were obtained from patients undergoing spinal surgery with patients’ written consent. AFC and NPC were isolated as described earlier [39] and hMSC were isolated using Histopaque-1077 (Sigma-Aldrich, Buchs, Switzerland) as previously described [42]. After expansion, the IVD cells and hMSC were plated in triplicates in well plates, and media supplemented with DMSO or DMSO/genipin (Table 2) was added to the cells. Cells were cultured for 14 days with media being refreshed every second to third day. At the set time points, mitochondrial activity was measured.

### 4.5. Mitochondrial Activity

Tissue samples of 382.20 ± 158.19 mg (mean ± SD) were taken next to the punch/repair site (injured) and from the contralateral side (intact). Samples were immersed in 50 µM resazurin sodium salt solution (Sigma-Aldrich) and incubated for 4 h at 37 °C. Relative fluorescence units (RFUs) were read on a plate reader (SpectraMax M5, Molecular Devices, distributed by Bucher Biotec, Basel, Switzerland) at an excitation wavelength of 547 nm and an emission wavelength of 582 nm. Read-outs were normalized to dry weight (dw) of respective tissue samples (50.10 ± 18.62 mg, mean ± SD) and further normalized to healthy control discs.

### 4.6. DNA Content

After determination of the mitochondrial activity, the samples were dried at 60 °C overnight, followed by papain digestion (3.9 U/mL, Sigma-Aldrich). Then, the DNA content was determined with Hoechst 3258 (Sigma-Aldrich) using a standard curve from calf thymus DNA (Sigma-Aldrich) and normalized to control IVDs. Fluorescence was measured at 350 nm excitation and 450 nm emission wavelength.

### 4.7. Extracellular Matrix Content

GAG content of the papain digested samples was determined by 1,9-dimethyl-methylene blue (Sigma-Aldrich) [43]. Absorbance was read at 600 nm on a plate reader, and GAG content was calculated with a standard curve from chondroitin sulphate (Sigma-Aldrich) and normalized to control IVDs.

### 4.8. Relative Gene Expression

Expression of multiple catabolic (*MMP3* and *MMP13*, *ADAMTS*4), anabolic (*ACAN*, *COL1*, *COL2*, *BGN*, *COMP*), and additionally, several inflammatory marker genes (*IL-1b*, *IL-8*, *CCL2*, *COX*2, and *NGF*) were analysed by using real-time reverse transcriptase quantitative polymerase chain reaction (qPCR) (Table 3). RNA was isolated from snap-frozen tissue samples, as described previously [30]. Residual DNA was degraded by DNase (DNase 1 Kit, Sigma-Aldrich, Buchs, Switzerland) and reverse transcription was performed with iScript™ cDNA synthesis Kit (Bio-Rad Inc., Cressier, Switzerland). iTaq™ universal SYBR^®^ Green supermix (Bio-Rad), forward and reverse primer (Microsynth, Balgach, Switzerland) for each gene, and cDNA were mixed to perform qPCR in duplicates (iQ5, Bio-Rad). 18S was used as the reference gene, and relative gene expression to healthy control IVDs was determined using the 2^−∆∆Ct^ method [44].

### 4.9. Histology

Cryosections were prepared after fixation of the IVDs in 4% formaldehyde solution, immersion in 15% and 30% sucrose followed by cartilaginous endplate removal and embedding in Tissue-Tek^®^ O.C.T.™ Compound (Sysmex, Horgen, Switzerland). Samples were then frozen in liquid nitrogen and cut into 16 µm transversal sections with a cryotome (microm HM560, Thermo Fisher Scientific, Basel, Switzerland). Poly(methyl methacrylate) (PMMA) embedded samples were dehydrated after fixation prior to their embedding in PMMA, and 6 µm sagittal sections were performed by using a microtome (microtome Leica 2155, Leica, Wetzlar, Germany). Sections were stained with Hematoxylin & Eosin (H&E) and with Safranin-O/Fast Green. Additionally, Picrosirius Red staining was performed on PMMA sections.

### 4.10. Statistics

The statistical significance for normally distributed data, i.e., disc height, mitochondrial activity, DNA, and GAG, was analysed by two-way ANOVA followed by Tukey’s multiple comparisons test. Significance was indicated by letters in graphs. Furthermore, one sample *t*-test with a hypothetical value of 0 was performed to determine the deviation from the control IVDs. Data are presented as mean ± SEM for an N = 5.

For gene expression data, a non-parametric distribution was assumed and presented as mean ± SEM for an N = 6. The data was analysed by Kruskal-Wallis tests and Dunn’s multiple pairwise comparison tests. Moreover, the Wilcoxon signed-rank test was calculated to test deviations from the hypothetical value of 1.

All tests were performed with GraphPad Prism version 6.0h (GraphPad Software, La Jolla, CA, USA).

## 5. Conclusions

In conclusion, the data presented here suggest that the combination of a genipin-enhanced fibrin hydrogel with the novel GMP-compliant engineered silk is an approach to repair AF injuries that might arise after IVD herniation surgery. The genipin-enhanced fibrin hydrogel acted successfully as a filler material, and the silk scaffold was able to form a barrier. In future studies, the population of this silk scaffold with IVD cells or MSC might be investigated in an organ culture setup. A further main question, which should be tackled, is whether cells are really needed to improve the outcome such as MSCs and/or AF cells pre-mixed with the hydrogel in this mechanobiological organ culture system [45].

## Figures and Tables

**Table 1:** Donor information of human mesenchymal stem cells (hMSC), annulus fibrosus (hAF) and nucleus pulposus (hNP) cells.

Cell Type	Gender	Age	Location	Passage
hMSC	Female	62	T1/2	3
hMSC	Male	86	L1/2	2
hMSC	Female	75	L3	3
hMSC	Female	60	L1-3	2
hMSC	Male	72	T8	2
hMSC	Female	69	T3-8	1
hAF & hNP	Male	47	T12/l1	2
hAF & hNP	Male	50	L2/3	2
hAF & hNP	Female	38	L5/6	2

**Table 2:** Overview of dimethyl sulfoxide (DMSO) and genipin concentrations used for cytotoxicity assay versus control medium (hMSC: α-MEM + 10% FCS; hAF/hNP: HG-DMEM + 10% FCS).

Medium	Abbreviation	DMSO (%)	Genipin (%)
Control	C		-
DMSO 1	D1	1.45	-
DMSO 2	D2	2.91	-
DMSO 3	D3	6.5	-
DMSO 4	D4	8.72	-
DMSO 5	D5	11.63	-
Genipin 1	G1	1.45	0.1
Genipin 2	G2	2.91	0.2
Genipin 3	G3	6.5	0.42
Genipin 4	G4	8.72	0.6
Genipin 5	G5	11.63	0.8

**Table 3:** Primers for gene expression used at an annealing temperature of 57 °C and a two-step protocol with 45 cycles.

Gene	Description	Forward Primer (5′-3′)	Reverse Primer (3′-5′)
18S	18S ribosomal RNA	ACG GAC AGG ATT GAC AGA TTG	CCA GAG TCT CGT TCG TTA TCG
ACAN	Aggrecan	GGC ATC GTG TTC CAT TAC AG	ACT CGT CCT TGT CTC CAT AG
COL1	Collagen type I alpha 2 chain	GCC TCG CTC ACC AAC TTC	AGT AAC CAC TGC TCC ATT CTG
COL2	Collagen type II alpha 1 chain	CGG GTG AAC GTG GAG AGA CA	GTC CAG GGT TGC CAT TGG AG
BGN	Biglycan	CTG CCA CTG CCA TCT GAG	TTG TTC ACG AGG ACC AAG G
COMP	Cartilage oligomeric matrix protein	TGC GAC GACGAC ATA CAC	ATC TCC TAC ACC ATC ACC ATC
MMP3	Matrix metallopeptidase 3	CTT CCG ATT CTG CTG TTG CTA TG	ATG GTG TCT TCC TTG TCC CTT G
MMP13	Matrix metallopeptidase 13	TCC TGG CTG GCT TCC TCT TC	CCT CGG ACA AGT CTT CAG AAT CTC
ADAMTS4	ADAM metallopeptidase with thrombospondin type 1 motif 4	GGC ACT GGG CTA CTA TTA C	TGG ACA CAG ACT GAG GAG
IL-1β	Interleukin 1 beta	AGT GCC ATC CTT CTG TCA	CAT TGC CTT CTC CGC TAT T
IL-8	Interleukin 8	CTT GTT CAA TAT GAC TTC CA	CCA CTC TCA ATA ACT CTC A
CCL2	Chemokine (C-C motif) ligand 2	TCG CCT GCT GCT ATA CAT T	TTG CTG CTG GTG ACT CTT
COX2	Cytochrome c oxidase subunit II	GGT AAT CCT ATA TGC TCT C	GTA TCT TGA ACA CTG AAT G
NGF	Nerve growth factor	ATG TTG TTC TAC ACT CTG	ATG CTG AAG TTT AAT CCA
